# Supplementary material for: The Evolution and Characterization of the RNA Interference Pathways in Lophotrochozoa
Source: Genome Biol Evol. 2024 May 7;16(5):evae098. doi: 10.1093/gbe/evae098 (PMC11114477; doi:10.1093/gbe/evae098)
Supplement: evae098_Supplementary_Data [file evae098_supplementary_data.zip › Supplementary_materials.zip!/selected_miRNAs/NoGe_selected_miRNAs/mirdeep_pdf_files/NMRB01000754.1_Notospermus_geniculatus_strain_Ushimado_scaffold754,_whole_genome_shotgun_sequence_20344.pdf]

Provisional ID	:	NMRB01000754.1_Notospermus_geniculatus_strain_Ushimado_scaffold754,_whole_genome_shotgun_sequence_20344
Score total	:	46794.8
Score for star read(s)	:	3.9
Score for read counts	:	46786.3
Score for mfe	:	1.7
Score for randfold	:	0
Score for cons. seed	:	3
Total read count	:	91781
Mature read count	:	91084
Loop read count	:	0
Star read count	:	697

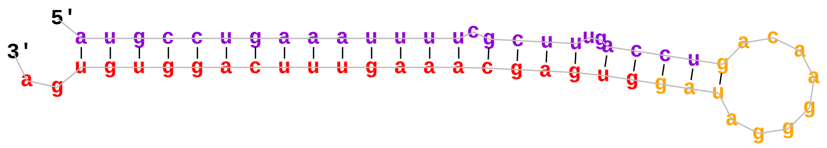

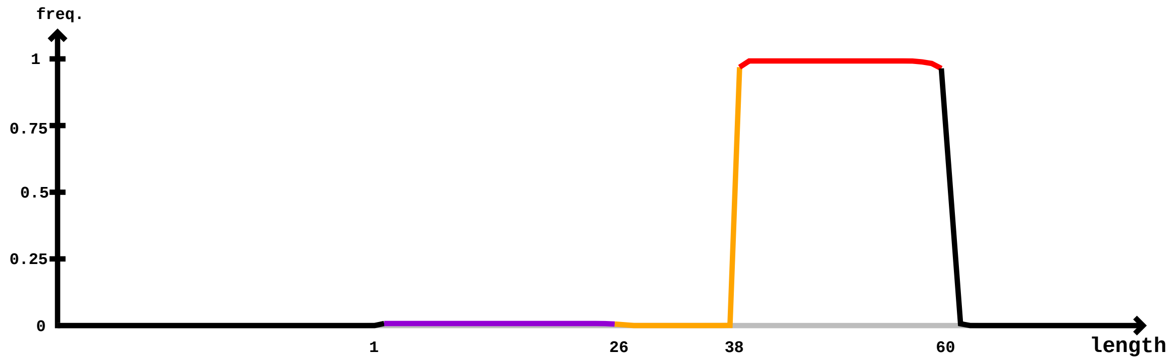

Star Mature

```
5'- uaggaguguaucuugacuugccguuguugguuaugccugaaauuuucgcuuugaccugacaagggauaggugagcaaaguuucaggugugacugccagguggccccaacac  -3'  obs
    uaggaguguaucuugacuugccguuguugguuaugccugaaauuuucgcuuugaccugacaagggauaggugagcaaaguuucaggugugacugccagguggccccaacac        exp
    (((((.....)))))....(((.(((.((((((((((((((((((.((((..(((((........))))))))))))))))))))))))))))).))).)))........       reads   mm   sample
    ...............................uaugccugaaauuuucgcuuugacc.......................................................       2       0    Mm3
    ................................augccugaaauuuucgcuuuga.........................................................       1       0    Mm3
    ................................augccugaaauuuucgcuuugac........................................................       5       0    Mm3
    ................................augccugaaauuuucgcuuugacc.......................................................       25      0    Mm3
    ................................augccugaaauuuucgcuuugacA......................................................       1       1    Mm3
    ................................augccugaaaAuuucgcuuugacc.......................................................       1       1    Mm3
    ................................augccugaaauuuucgcCuugaccu......................................................       2       1    Mm3
    ................................augccugaaauuuucgcuuugaccu......................................................       128     0    Mm3
    ................................augccugaaauuuucgcuuugaccG......................................................       5       1    Mm3
    ................................augccugaaauuuucgAuuugaccu......................................................       1       1    Mm3
    ................................Nugccugaaauuuucgcuuugaccu......................................................       1       1    Mm3
    ................................augccugaaauuuucgcuuugaccuU.....................................................       2       1    Mm3
    ................................augccugaaauuuucgcuuugaccug.....................................................       1       0    Mm3
    ...................................................................uGggugagcaaaguuucaggugugа....................       1       1    Mm3
    ....................................................................Cgugagcaaaguuucaggugugа....................       1       1    Mm3
    .....................................................................gugagcaaaguuucaggu........................       1       0    Mm3
    .....................................................................gugagcaaaguuucaggug.......................       7       0    Mm3
    .....................................................................gugagcaaaguuucaggGgu......................       1       1    Mm3
    .....................................................................gugagcaaaguuucaggugG......................       1       1    Mm3
    .....................................................................gugagcaaaguuucaggugu......................       45      0    Mm3
    .....................................................................gugagcaaaguuucaggugA......................       1       1    Mm3
    .....................................................................gugagcaaaguuucaggCgu......................       1       1    Mm3
    .....................................................................Nugagcaaaguuucaggugug.....................       1       1    Mm3
    .....................................................................gugagUaaaguuucaggugug.....................       1       1    Mm3
    .....................................................................gugagcaaaguuucaggAgug.....................       1       1    Mm3
    .....................................................................gugagcaaaguuucaggugug.....................       100     0    Mm3
    .....................................................................gugagcaaagAuucaggugug.....................       1       1    Mm3
    .....................................................................gugagNaaaguuucaggugugа....................       1       1    Mm3
    .....................................................................gugagcaaaguuucaggCguga....................       23      1    Mm3
    .....................................................................gugagcaaaguuucaggugugG....................       709     1    Mm3
    .....................................................................gugagcaaagCuucaggugugа....................       2       1    Mm3
    .....................................................................gugagcaaCguuucaggugugа....................       1       1    Mm3
    .....................................................................guUagcaaaguuucaggugugа....................       1       1    Mm3
```

Star **Mature**

```
uaggaguguaucuugacuugccguuguugguuaugccugaaauuuucgcuuugaccugacaagggauaggugagcaaaguuucagguguga cugccagguggccccaacac
.....................................................................Uugagcaaaguuucagguguga....................  2     1   Mm3
.....................................................................Nugagcaaaguuucagguguga....................  1     1   Mm3
.....................................................................gugagcaaaguuucaggugugC....................  4     1   Mm3
.....................................................................gugagcaCaguuucagguguga....................  3     1   Mm3
.....................................................................gCgagcaaaguuucagguguga....................  1     1   Mm3
.....................................................................gugagcaaaguuucaggGguga....................  14    1   Mm3
.....................................................................gugagcaaaguuucagguguUa....................  4     1   Mm3
.....................................................................gugagcaaaguuucGgguguga....................  2     1   Mm3
.....................................................................gugagcaaaguuCcagguguga....................  1     1   Mm3
.....................................................................gugagcaaaguuucagguUuga....................  2     1   Mm3
.....................................................................gugagcaaaguuucUgguguga....................  1     1   Mm3
.....................................................................guAagcaaaguuucagguguga....................  1     1   Mm3
.....................................................................gAgagcaaaguuucagguguga....................  3     1   Mm3
.....................................................................gugagcaaaguuucaUguguga....................  1     1   Mm3
.....................................................................gugGgcaaaguuucagguguga....................  2     1   Mm3
.....................................................................gugagcaaagAuucagguguga....................  8     1   Mm3
.....................................................................gugUgcaaaguuucagguguga....................  2     1   Mm3
.....................................................................gugagcaaaguuucaggAguga....................  27    1   Mm3
.....................................................................gGgagcaaaguuucagguguga....................  2     1   Mm3
.....................................................................gugagcaaaguuucagUuguga....................  4     1   Mm3
.....................................................................gugagcaaaguuucagAuguga....................  13    1   Mm3
.....................................................................gugagcaaaguCucagguguga....................  1     1   Mm3
.....................................................................gugagcaaaguuuUagguguga....................  2     1   Mm3
.....................................................................gugagcGaaguuucagguguga....................  1     1   Mm3
.....................................................................gugagcaaaguuucaggugAga....................  1     1   Mm3
.....................................................................gugagcaaaguuucagguguga....................  5121  0   Mm3
.....................................................................gugagcUaaguuucagguguga....................  1     1   Mm3
.....................................................................gugagcaaaguuucaggugugU....................  153   1   Mm3
.....................................................................gugagcaaaguuucagCuguga....................  15    1   Mm3
.....................................................................gugaUcaaaguuucagguguga....................  1     1   Mm3
.....................................................................gugCgcaaaguuucagguguga....................  2     1   Mm3
.....................................................................gugagcaaagGuucagguguga....................  9     1   Mm3
.....................................................................gugagcaaaguuucaggugugac...................  15    0   Mm3
.....................................................................gugagcaaaguuucaggugugaU...................  178   1   Mm3
.....................................................................gugagcaaaguuucaggugugaA...................  271   1   Mm3
.....................................................................gugagcaaaguuucaggugugaG...................  4     1   Mm3
.....................................................................gugagcaaaguuucaggugugaUu..................  29    1   Mm3
.....................................................................gugagcaaaguuucaggugugaGu..................  2     1   Mm3
.....................................................................gugagcaaaguuucaggugugaAu..................  17    1   Mm3
.....................................................................gugagcaaaguuucaggugugacu..................  2     0   Mm3
......................................................................ugagcaaaguuucaggugug.....................  2     0   Mm3
......................................................................ugagcaaaguuucaggugugG....................  12    1   Mm3
......................................................................ugagcaaaguGucagguguga....................  1     1   Mm3
......................................................................ugagcaaaguuucagguguga....................  75    0   Mm3
......................................................................ugagcaaaguuucagguUuga....................  1     1   Mm3
......................................................................ugagcaaaguuucaggugugU....................  2     1   Mm3
......................................................................ugagcaaaguuucagguAuga....................  2     1   Mm3
......................................................................ugagcaaUguuucaggugugac...................  1     1   Mm3
......................................................................ugagcaaaguuucaggugugaA...................  7     1   Mm3
......................................................................ugagcaaaguuucaggugugac...................  4     0   Mm3
......................................................................ugagcaaaguuucaggugugaU...................  8     1   Mm3
......................................................................ugagcaaaguuucaggugugacu..................  1     0   Mm3
......................................................................ugagcaaaguuucaggugugacA..................  1     1   Mm3

............uugacuugccguuguugguu...............................................................................  3     0   Fm3
...............................uaugccugaaauuuucgcuuugacc.......................................................  2     0   Fm3
................................augccugaaauuuucgcuuugac........................................................  2     0   Fm3
................................augccugaaauuuucgcuuugacc.......................................................  14    0   Fm3
................................augccugaaauuuucgcuuugaccu......................................................  68    0   Fm3
................................augccugaaauuuucgcGuugaccu......................................................  1     1   Fm3
................................augccugaaaAuuucgcuuugaccu......................................................  1     1   Fm3
....................................................................Cgugagcaaaguuucagguguga....................  12    1   Fm3
.....................................................................gugagcaaaguuucaggu........................  1     0   Fm3
.....................................................................gugagcaaaguuucagguA.......................  1     1   Fm3
.....................................................................gugagcaaaguuucaggug.......................  57    0   Fm3
.....................................................................gugagcaaaguuucaggAgu......................  2     1   Fm3
.....................................................................gugagcaaaguuucaggugu......................  84    0   Fm3
.....................................................................gugagcaaaguuucaggugA......................  3     1   Fm3
.....................................................................gugagcaaaguuucaggGgu......................  1     1   Fm3
.....................................................................gugagcaaaguuucagCugug.....................  2     1   Fm3
```

```
                                Star                                Mature
uaggaguguaucuugacuugccguuguugguuaugccugaaauuuucgcuuugaccugacaagggauaggugagcaaaguuucaggugugacugccagguggccccaacac
.....................................................................gugagcGaaguuucaggugug.....................   1       1   Fm3
.....................................................................gugagcaaagAuucaggugug.....................   1       1   Fm3
.....................................................................gugagcaaaguuucaggugug.....................   549     0   Fm3
.....................................................................gugagcaaaguuuAaggugug.....................   1       1   Fm3
.....................................................................gugagNaaaguuucaggugug.....................   1       1   Fm3
.....................................................................gugagcaaaguuucaggCgug.....................   5       1   Fm3
.....................................................................gugagcaaGguuucaggugug.....................   1       1   Fm3
.....................................................................gugagcaaaguuucaggAgug.....................   2       1   Fm3
.....................................................................gugagcaaaguuucagUugug.....................   2       1   Fm3
.....................................................................gugagcaaaguuucaggGgug.....................   3       1   Fm3
.....................................................................gugagcUaaguuucaggugug.....................   1       1   Fm3
.....................................................................gugUgcaaaguuucaggugugа....................   5       1   Fm3
.....................................................................guUagcaaaguuucaggugugа....................   4       1   Fm3
.....................................................................gugagcUaaguuucaggugugа....................   6       1   Fm3
.....................................................................gugagcaaaguuucaggCguga....................   219     1   Fm3
.....................................................................gugagcaaaguuucaggugGga....................   4       1   Fm3
.....................................................................gugagcaaaguuucagAuguga....................   72      1   Fm3
.....................................................................gCgagcaaaguuucaggugugа....................   3       1   Fm3
.....................................................................gugagcaaaguuucGgguguga....................   3       1   Fm3
.....................................................................gugGgcaaaguuucaggugugа....................   4       1   Fm3
.....................................................................gugagcaaaguuCcaggugugа....................   2       1   Fm3
.....................................................................gugagcCaaguuucaggugugа....................   1       1   Fm3
.....................................................................Cugagcaaaguuucaggugugа....................   1       1   Fm3
.....................................................................gugagcaaaguuucagguguUa....................   5       1   Fm3
.....................................................................gugagcaaaguuucagUuguga....................   36      1   Fm3
.....................................................................gugagNaaaguuucaggugugа....................   5       1   Fm3
.....................................................................gugagcaaaguuAcaggugugа....................   1       1   Fm3
.....................................................................gugagcaaaAuuucaggugugа....................   1       1   Fm3
.....................................................................gugagcaaaguuucaggAguga....................   171     1   Fm3
.....................................................................gugagcaGaguuucaggugugа....................   2       1   Fm3
.....................................................................gugagcaaaguuucaggugugа....................   29819   0   Fm3
.....................................................................gugaAcaaaguuucaggugugа....................   3       1   Fm3
.....................................................................gugagGaaaguuucaggugugа....................   2       1   Fm3
.....................................................................gugagcaaaguuuUaggugugа....................   34      1   Fm3
.....................................................................Nugagcaaaguuucaggugugа....................   14      1   Fm3
.....................................................................gugagcaaaguuGcaggugugа....................   1       1   Fm3
.....................................................................gugagcaaGguuucaggugugа....................   3       1   Fm3
.....................................................................gugagcaaaguuucaggugAga....................   7       1   Fm3
.....................................................................gugagcaaaguuucagguCuga....................   1       1   Fm3
.....................................................................gugaUcaaaguuucaggugugа....................   2       1   Fm3
.....................................................................gugagcaaaguuucaggugugC....................   14      1   Fm3
.....................................................................gugagcaaagAuucaggugugа....................   60      1   Fm3
.....................................................................Augagcaaaguuucaggugugа....................   3       1   Fm3
.....................................................................gugagcaaaguuucaggugugG....................   4       1   Fm3
.....................................................................gugagcaaaguuucagguUuga....................   2       1   Fm3
.....................................................................gugagUaaaguuucaggugugа....................   3       1   Fm3
.....................................................................gugagcaaCguuucaggugugа....................   8       1   Fm3
.....................................................................gAgagcaaaguuucaggugugа....................   14      1   Fm3
.....................................................................gugagcGaaguuucaggugugа....................   5       1   Fm3
.....................................................................gugCgcaaaguuucaggugugа....................   10      1   Fm3
.....................................................................gugagcaaaguuucUggugugа....................   1       1   Fm3
.....................................................................gugagcaaaguuucaUguguga....................   12      1   Fm3
.....................................................................gugagcaaaguuucCgguguga....................   1       1   Fm3
.....................................................................gugagcaaagGuucaggugugа....................   52      1   Fm3
.....................................................................guCagcaaaguuucaggugugа....................   3       1   Fm3
.....................................................................gugagcaaaguuucaggGguga....................   127     1   Fm3
.....................................................................gugagcaaagCuucaggugugа....................   40      1   Fm3
.....................................................................gugagcaaaguuucagguAuga....................   6       1   Fm3
.....................................................................gugagcaaaguCucaggugugа....................   3       1   Fm3
.....................................................................gugagcaaaguuucagguguAa....................   6       1   Fm3
.....................................................................gugagcaaaguuucaggugugU....................   1002    1   Fm3
.....................................................................gGgagcaaaguuucaggugugа....................   4       1   Fm3
.....................................................................gugagcaaaUuuucaggugugа....................   3       1   Fm3
.....................................................................gugagcaaaguuuAaggugugа....................   8       1   Fm3
.....................................................................gugagcaaaguuucaCguguga....................   1       1   Fm3
.....................................................................gugagcaaaguuucaggugCga....................   4       1   Fm3
.....................................................................gugagcaUaguuucaggugugа....................   14      1   Fm3
.....................................................................gugagcaaaguuucagguguCa....................   2       1   Fm3
.....................................................................gugagcaaaguuucaAguguga....................   1       1   Fm3
.....................................................................gugagcaaaguuGaggugugа....................   2       1   Fm3
```

Star **Mature**

uaggaguguaucuugacuugccguuguugguuaugccugaaauuuucgcuuugaccugacaagggauaggugagcaaaguuucaggugugacugccaggugccccaacac

....gugagcaaaguuucagCuguga...................	87	1	Fm3
....gugagcaCaguuucaggugugа...................	7	1	Fm3
....Uugagcaaaguuucaggugugа...................	12	1	Fm3
....gugaCcaaaguuucaggugugа...................	5	1	Fm3
....guAagcaaaguuucaggugugа...................	10	1	Fm3
....gugagAaaaguuucaggugugа...................	2	1	Fm3
....gugagcaaaguuucaggugugaG..................	13	1	Fm3
....gugagcaaaguuucaggCgugac..................	1	1	Fm3
....gugagcaaaguuucaggugugac..................	33	0	Fm3
....gugagcaaaguuucaggugugaA..................	1013	1	Fm3
....gugagcaaaguuucaggugugaU..................	626	1	Fm3
....gugagcaaUguuucaggugugac..................	12	1	Fm3
....gugagcaaaguuucagguCugac..................	1	1	Fm3
....gugagcaaaguuucaggugugCcu.................	1	1	Fm3
....gugagcaaaguuucaggugugaUu.................	47	1	Fm3
....gugagcaaaguuucaggugugacu.................	3	0	Fm3
....gugagcaaaguuucaggugugaGu.................	8	1	Fm3
....gugagcaaaguuucaggugugaAu.................	19	1	Fm3
....ugagcaaaguuucaggug......................	1	0	Fm3
....ugagcaaaguuucaggugu.....................	1	0	Fm3
....ugagcaaaguuucaggugug....................	4	0	Fm3
....ugagcaaaguuucGggugugа...................	1	1	Fm3
....ugagcaaaguuucagguCuga...................	7	1	Fm3
....ugagcaaaguuucaggAguga...................	4	1	Fm3
....uUagcaaaguuucaggugugа...................	1	1	Fm3
....ugagUaaaguuucaggugugа...................	1	1	Fm3
....ugagcaaaguuucaggCguga...................	1	1	Fm3
....ugagcaaaguuucUggugugа...................	1	1	Fm3
....ugagcaaaguuucagguguAa...................	1	1	Fm3
....ugagcaaaguuucaggugugC...................	2	1	Fm3
....ugagcaaaguuucagguUuga...................	4	1	Fm3
....ugCgcaaaguuucaggugugа...................	1	1	Fm3
....ugagcaaaguuucagCuguga...................	1	1	Fm3
....ugagcaaaguuucaggugugU...................	65	1	Fm3
....uAagcaaaguuucaggugugа...................	1	1	Fm3
....ugagcaaaguGucaggugugа...................	5	1	Fm3
....ugagcaaaguAucaggugugа...................	4	1	Fm3
....ugUgcaaaguuucaggugugа...................	1	1	Fm3
....ugagcaaaguuucagguAuga...................	8	1	Fm3
....ugagcaaaguuucaggugugа...................	918	0	Fm3
....ugagcaaaguuucaggugugac..................	32	0	Fm3
....ugagcaaaguuucaggugugaA..................	112	1	Fm3
....ugagcaaaguuucaggugugaU..................	97	1	Fm3
....ugagcaaUguuucaggugugac..................	15	1	Fm3
....ugagcaaaguuucagguUugac..................	1	1	Fm3
....ugagcaaaguuucaggugugaUu.................	3	1	Fm3
....ugagcaaaguuucaggugugaAu.................	2	1	Fm3
....ugagcaaUguuucaggugugacu.................	6	1	Fm3
....ugagcaaaguuucaggugugacu.................	6	0	Fm3
....gagcaaaguuucaggugugа...................	1	0	Fm3
...............................uaugccugaaauuuucgcuuugac...	1	0	Mm2
...............................uaugccugaaauuuucgcuuugacc..	1	0	Mm2
................................augccugaaauuuucgcuuugU..	1	1	Mm2
................................augccugaaauuuucgcuuugac...	4	0	Mm2
................................augccugaaaAuuucgcuuugac...	1	1	Mm2
................................augccugaaauuuucgcuuugacc..	18	0	Mm2
................................Uugccugaaauuuucgcuuugaccu...	1	1	Mm2
................................augccugaGauuuucgcuuugaccu...	1	1	Mm2
................................augccugaaauuuucgcuuugaccA..	1	1	Mm2
................................augccugaaauuuucgcuuugaccu...	84	0	Mm2
................................augccugaaauuuucgcCuugaccu...	1	1	Mm2
................................augccugaaauuuucgcuuugaccG..	5	1	Mm2
................................augccugaaauuuucgcuuugaccuU...	2	1	Mm2
................................augccugaaauuuucgcAuugaccugacaagggauag...	1	1	Mm2
....Cgugagcaaaguuucaggugugа...................	5	1	Mm2
....gugagcaaaguuucaggu.......................	5	0	Mm2
....gugagcaaaguuucagguA......................	1	1	Mm2
....gugagcaaaguuucaggug......................	31	0	Mm2
....gCgagcaaaguuucaggugu.....................	2	1	Mm2

Star Mature

uaggaguguaucuugacuugccguuguugguuaugccugaaauuuucgcuuugaccugacaagggauaggugagcaaaguuucaggugugacugccaggugccccaacac			
....gugagcaaaguuucaggCgu.....................	2	1	Mm2
....gugagcaaaguuucaggugG.....................	2	1	Mm2
....gugagcaaaguuucagCugu.....................	1	1	Mm2
....gugagcaaaguuucaggugu.....................	49	0	Mm2
....gugagcaaaguuucagguguU....................	2	1	Mm2
....gugagcaaaguuucaggCgug....................	3	1	Mm2
....gugagcaaaguuucagUugug....................	1	1	Mm2
....gugagcaaaguuucaggAgug....................	2	1	Mm2
....gugagcaaaguuucaggugug....................	154	0	Mm2
....gugagcaaaguuucaggGgug....................	2	1	Mm2
....gugagcaaaguuuAaggugugа...................	2	1	Mm2
....gugagcaaagAuucaggugugа...................	14	1	Mm2
....gugagcaaaguuucagguUuga...................	1	1	Mm2
....gugagcaaaguuucaggGguga...................	26	1	Mm2
....gugagcaaaguuucaggugugG...................	1765	1	Mm2
....gugagcCaaguuucaggugugа...................	1	1	Mm2
....gugagcaaaguuucagguAuga...................	1	1	Mm2
....gugagAaaaguuucaggugugа...................	1	1	Mm2
....gugagcaaaguuucaggugugа...................	7398	0	Mm2
....gugagcaaagGuucaggugugа...................	7	1	Mm2
....gugagcaCaguuucaggugugа...................	1	1	Mm2
....Uugagcaaaguuucaggugugа...................	3	1	Mm2
....gugagcaaCguuucaggugugа...................	2	1	Mm2
....gugaCcaaaguuucaggugugа...................	1	1	Mm2
....gugagcaaaguuucaCguguga...................	2	1	Mm2
....gugagNaaaguuucaggugugа...................	1	1	Mm2
....Nugagcaaaguuucaggugugа...................	4	1	Mm2
....gugagcUaaguuucaggugugа...................	2	1	Mm2
....gugagcaaagCuucaggugugа...................	4	1	Mm2
....Augagcaaaguuucaggugugа...................	3	1	Mm2
....gugagcaaaguuUaggugugа...................	6	1	Mm2
....gugagcaaaguuucaggugugC...................	12	1	Mm2
....gugaUcaaaguuucaggugugа...................	1	1	Mm2
....gugagcaaaguuucaggugCga...................	2	1	Mm2
....gCgagcaaaguuucaggugugа...................	2	1	Mm2
....gugagcaaaguuucagUuguga...................	9	1	Mm2
....gAgagcaaaguuucaggugugа...................	4	1	Mm2
....gugCgcaaaguuucaggugugа...................	2	1	Mm2
....gugagcaaaguuucagAuguga...................	19	1	Mm2
....gugagUaaaguuucaggugugа...................	2	1	Mm2
....gGgagcaaaguuucaggugugа...................	1	1	Mm2
....gugagcaaaguuucGggugugа...................	3	1	Mm2
....gugagcaaaguuCcaggugugа...................	1	1	Mm2
....gugagcaGaguuucaggugugа...................	1	1	Mm2
....gugagcaaaguuucagCuguga...................	20	1	Mm2
....gugagcaaaguuucaggAguga...................	52	1	Mm2
....gugagcaaaguuucaggugugU...................	183	1	Mm2
....gugagcGaaguuucaggugugа...................	1	1	Mm2
....gugagcaUaguuucaggugugа...................	2	1	Mm2
....gugagcaaaguuucaggCguga...................	53	1	Mm2
....gugagcaaaguuucaggugugac..................	18	0	Mm2
....gugUgcaaaguuucaggugugac..................	1	1	Mm2
....gugagcaaaguuucaggugugaA..................	500	1	Mm2
....gugagcaaaguuucaggAgugac..................	1	1	Mm2
....gugagcaaaguuucaggugugaG..................	6	1	Mm2
....gugagcaaaguuucaggugugaU..................	296	1	Mm2
....gugagcaaaguuucaggugugaUu.................	37	1	Mm2
....gugagcaaaguuucaggugugacu.................	5	0	Mm2
....gugagcaaaguuucaggugugaAu.................	14	1	Mm2
....gugagcaaaguuucaggugugacA.................	2	1	Mm2
....gugagcaaaguuucaggugugaGu.................	5	1	Mm2
....gugagcaaaguuucaggugugacC.................	6	1	Mm2
....gugagcaaaguuucaggugugacuU................	1	1	Mm2
....ugagcaaaguuucaggugug....................	1	0	Mm2
....ugagcaaaguuucaggugugG...................	25	1	Mm2
....ugagcaaaguuucagguAuga...................	3	1	Mm2
....ugagcaaaguuucagguUuga...................	1	1	Mm2
....ugagcaaaguuucagguguga...................	92	0	Mm2
....ugagcaaaguuucaggugugU...................	5	1	Mm2
....ugagcaaaguuucaggugugA...................	8	1	Mm2

```
                                Star                                  Mature
uaggaguguaucuugacuugccguuguugguuaugccugaaauuuucgcuuugaccugacaagggauaggugagcaaaguuucaggugugacugccagguggccccaacac
.......................................................................ugagcaaaguuucaggugugaG...................   1      1    Mm2
.......................................................................ugagcaaaguuucaggugugac...................   12     0    Mm2
.......................................................................ugagcaaaguuucaggugugaU...................   16     1    Mm2
.......................................................................ugagcaaUguuucaggugugac...................   1      1    Mm2
.......................................................................ugagcaaaguuucaggugugaAu..................   1      1    Mm2
.......................................................................ugagcaaaguuucaggugugacu..................   1      0    Mm2
.......................................................................ugagcaaUguuucaggugugacu..................   2      1    Mm2
.......................................................................ugagcaaaguuucaggugugaUu..................   1      1    Mm2

................................augccugaaauuuucgcuuugacc.......................................................   3      0    Mm1
................................augccugaaauuuucgcuuugaccu......................................................   5      0    Mm1
................................augccugaaauuuucgcuuugaccugacaagggauagU.........................................   1      1    Mm1
.....................................................................gAgagcaaaguuucaggugu......................   1      1    Mm1
.....................................................................gugagcaaaguuucaggugu......................   15     0    Mm1
.....................................................................gugagcaaagGuucaggugug.....................   1      1    Mm1
.....................................................................gugagcaaaguuucaggugug.....................   17     0    Mm1
.....................................................................gAgagcaaaguuucaggugugа....................   1      1    Mm1
.....................................................................guAagcaaaguuucaggugugа....................   2      1    Mm1
.....................................................................gGgagcaaaguuucaggugugа....................   1      1    Mm1
.....................................................................gugagcaaaguuucaggugugU....................   97     1    Mm1
.....................................................................Uugagcaaaguuucaggugugа....................   4      1    Mm1
.....................................................................gugagcaaaguuuUaggugugа....................   1      1    Mm1
.....................................................................gugagcaaaguuucaggAguga....................   15     1    Mm1
.....................................................................gugaUcaaaguuucaggugugа....................   2      1    Mm1
.....................................................................gugagcaaaguuucagUuguga....................   4      1    Mm1
.....................................................................gugUgcaaaguuucaggugugа....................   1      1    Mm1
.....................................................................gugagcaaaguuucaCguguga....................   1      1    Mm1
.....................................................................gugagcaaagAuucaggugugа....................   3      1    Mm1
.....................................................................gugagcaaaguuucaggugugG....................   130    1    Mm1
.....................................................................gugagcaaaguuucagguguUa....................   1      1    Mm1
.....................................................................gugagcaaaguuucaggGguga....................   12     1    Mm1
.....................................................................gugagcaaagGuucaggugugа....................   5      1    Mm1
.....................................................................gugagcaaaguuucaggugugа....................   3084   0    Mm1
.....................................................................gugagcaaaguuucagAuguga....................   7      1    Mm1
.....................................................................gugagcaaaguuucGgguguga....................   2      1    Mm1
.....................................................................gugagcaaaguuGaggugugа....................   1      1    Mm1
.....................................................................gugGgcaaaguuucaggugugа....................   1      1    Mm1
.....................................................................gugagcaCaguuucaggugugа....................   2      1    Mm1
.....................................................................gugagcaaaguuucaUguguga....................   1      1    Mm1
.....................................................................gugagcaaaguuucCgguguga....................   1      1    Mm1
.....................................................................gugagcaaaguuucaggCguga....................   12     1    Mm1
.....................................................................gugagcaaagCuucaggugugа....................   3      1    Mm1
.....................................................................gugagcaaaguuucagguUuga....................   2      1    Mm1
.....................................................................gugagcaGaguuucaggugugа....................   1      1    Mm1
.....................................................................gugagcUaaguuucaggugugа....................   1      1    Mm1
.....................................................................gugagAaaaguuucaggugugа....................   1      1    Mm1
.....................................................................Nugagcaaaguuucaggugugа....................   2      1    Mm1
.....................................................................gugagcaaaguuucagCuguga....................   8      1    Mm1
.....................................................................gugagcaUaguuucaggugugа....................   1      1    Mm1
.....................................................................gugagcaaaguuucaggugugaG...................   6      1    Mm1
.....................................................................gugagcaaaguuucaggugugaU...................   77     1    Mm1
.....................................................................gugagcaaaguuucaggugugaA...................   38     1    Mm1
.....................................................................gugagcaaaguuucaggugugaAu..................   23     1    Mm1
......................................................................ugagcaaaguuucaggugugG....................   2      1    Mm1
......................................................................ugagcaaaguuucaggugugа....................   22     0    Mm1
......................................................................ugagcaaaguuucagguAuga....................   1      1    Mm1

................................augccugaaauuuucgcuuugac........................................................   15     0    Fm2
................................augccugaaauuuucgcuuugacc.......................................................   72     0    Fm2
................................augccugaaauuuucgcuuugaccA......................................................   1      1    Fm2
................................augccugaaauuuucgcCuugaccu......................................................   1      1    Fm2
................................augccugaaauuuucgcuuugaccG......................................................   10     1    Fm2
................................augccugaaauuuucgAuuugaccu......................................................   2      1    Fm2
................................augccugaaauuuucgcuuugaccu......................................................   110    0    Fm2
................................Gugccugaaauuuucgcuuugaccu......................................................   1      1    Fm2
................................augccugaaauuuucgGuuugaccu......................................................   1      1    Fm2
................................augccugaaauuuucgcuuugaccuU.....................................................   17     1    Fm2
................................augccugaaauuuucgcuuugaccugacaagggauagA.........................................   11     1    Fm2
....................................................................Cgugagcaaaguuucaggugugа....................   11     1    Fm2
.....................................................................gugagcaaaguuucaggu........................   2      0    Fm2
```

```
                                Star                                Mature
uaggaguguaucuugacuugccguuguugguuaugccugaaauuuucgcuuugaccugacaagggauaggugagcaaaguuucaggugugacugccaggugggccccaacac
.....................................................................gugagcaaaguuucagguA........................   5      1    Fm2
.....................................................................gugagcaaaguuucaggAg........................   1      1    Fm2
.....................................................................gugagcaaaguuucagguU........................   3      1    Fm2
.....................................................................gugagcaaaguuucaggug........................   28     0    Fm2
.....................................................................gugagcaaaguuucaggugu.......................   63     0    Fm2
.....................................................................gugagcaaaguuucaggugG.......................   5      1    Fm2
.....................................................................gugagcaaaguuucaggAgu.......................   1      1    Fm2
.....................................................................gugagcaaaguuucaggAgug......................   1      1    Fm2
.....................................................................gugagcaaaguuucaggugug......................   287    0    Fm2
.....................................................................gugagcaaaguuucaggCgug......................   1      1    Fm2
.....................................................................gugagcaaagAuucaggugug......................   3      1    Fm2
.....................................................................gugagcaaaguuucaggGgug......................   1      1    Fm2
.....................................................................gugagcaaaguuCcagguguga.....................   2      1    Fm2
.....................................................................gugagcaaaguuucagguAuga.....................   1      1    Fm2
.....................................................................gugagcaaaguuucaggCguga.....................   41     1    Fm2
.....................................................................guUagcaaaguuucagguguga.....................   3      1    Fm2
.....................................................................Uugagcaaaguuucagguguga.....................   7      1    Fm2
.....................................................................gugagcaaaguCucagguguga.....................   1      1    Fm2
.....................................................................Nugagcaaaguuucagguguga.....................   2      1    Fm2
.....................................................................gugagcaaaguuucagguguAa.....................   1      1    Fm2
.....................................................................guCagcaaaguuucagguguga.....................   3      1    Fm2
.....................................................................gugagcaaagGuucagguguga.....................   4      1    Fm2
.....................................................................gugagcaaaguuucaggAguga.....................   43     1    Fm2
.....................................................................gugUgcaaaguuucagguguga.....................   2      1    Fm2
.....................................................................Augagcaaaguuucagguguga.....................   1      1    Fm2
.....................................................................gugagcaaaguuucaggugugU.....................   200    1    Fm2
.....................................................................gugagcaaaguuucaggugCga.....................   2      1    Fm2
.....................................................................gugCgcaaaguuucagguguga.....................   2      1    Fm2
.....................................................................gugagcGaaguuucagguguga.....................   1      1    Fm2
.....................................................................gugagAaaaguuucagguguga.....................   1      1    Fm2
.....................................................................gugagcaaaguuucaggGguga.....................   40     1    Fm2
.....................................................................guAagcaaaguuucagguguga.....................   5      1    Fm2
.....................................................................gugagcaaaguuucaggguUa.....................   7      1    Fm2
.....................................................................gAgagcaaaguuucagguguga.....................   1      1    Fm2
.....................................................................gugagcaaGguuucagguguga.....................   1      1    Fm2
.....................................................................gugagcaaaguuucagUuguga.....................   11     1    Fm2
.....................................................................gugagcaUaguuucagguguga.....................   2      1    Fm2
.....................................................................gugagcaaCguuucagguguga.....................   2      1    Fm2
.....................................................................gugagcaaaguuucUgguguga.....................   1      1    Fm2
.....................................................................gugagcaaaguuucaggugugG.....................   2130   1    Fm2
.....................................................................gugagcaCaguuucagguguga.....................   2      1    Fm2
.....................................................................gugaAcaaaguuucagguguga.....................   1      1    Fm2
.....................................................................gugagcaaaguuucGgguguga.....................   6      1    Fm2
.....................................................................gugagcaaaguuGcagguguga.....................   1      1    Fm2
.....................................................................gugagcaGaguuucagguguga.....................   3      1    Fm2
.....................................................................gugagcaaaguuucaggugugC.....................   11     1    Fm2
.....................................................................gugGgcaaaguuucagguguga.....................   4      1    Fm2
.....................................................................gugagcaaagCuucagguguga.....................   5      1    Fm2
.....................................................................gugagcaaaguuucagAuguga.....................   16     1    Fm2
.....................................................................gugagcaaaguuucagguguga.....................   7706   0    Fm2
.....................................................................Cugagcaaaguuucagguguga.....................   1      1    Fm2
.....................................................................gugagcaaagAuucagguguga.....................   15     1    Fm2
.....................................................................gugagcaaaguuucagCuguga.....................   21     1    Fm2
.....................................................................gugagcaaaguuucaUguguga.....................   1      1    Fm2
.....................................................................gugagNaaaguuucagguguga.....................   4      1    Fm2
.....................................................................gugagcaaUguuucaggugugac....................   8      1    Fm2
.....................................................................gugagcaaaguuucaggGgugac....................   1      1    Fm2
.....................................................................gugagcaaaguuucaggugugaU....................   360    1    Fm2
.....................................................................gugagcaaaguuucaggugugac....................   20     0    Fm2
.....................................................................gugagcaaaguuucaggugugaA....................   539    1    Fm2
.....................................................................gugagcaaaguuucaggugugaG....................   11     1    Fm2
.....................................................................gugagcaaaguuucaggugugaGu...................   22     1    Fm2
.....................................................................gugagcaaaguuucaggugugaAu...................   30     1    Fm2
.....................................................................gugagcaaaguuucaggugugaUu...................   61     1    Fm2
.....................................................................gugagcaaaguuucaggugugacG...................   1      1    Fm2
.....................................................................gugagcaaaguuucaggugugacC...................   17     1    Fm2
.....................................................................gugagcaaaguuucaggCgugacu...................   1      1    Fm2
.....................................................................gugagcaaaguuucaggugugacu...................   14     0    Fm2
......................................................................ugagcGaaguuucagguguga.....................   1      1    Fm2
......................................................................ugagcaaaguuucagguguga.....................   116    0    Fm2
```

```
                                Star                                Mature
uaggaguguaucuugacuugccguuguugguuaugccugaaauuuucgcuuugaccugacaagggauaggugagcaaaguuucaggugugacugccaggugccccaacac
......................................................................ugagcaaaguuucaggugugU...................   7     1   Fm2
......................................................................ugagcaaaguuucaggugugG...................   35    1   Fm2
......................................................................ugagcaaagCuucagguguga...................   1     1   Fm2
......................................................................ugagcaaaguuucaggugugac..................   22    0   Fm2
......................................................................ugagcaaaguuucaggugugaU..................   20    1   Fm2
......................................................................ugagcaaUguuucaggugugac..................   7     1   Fm2
......................................................................ugagcaaaguuucaggugugacu.................   27    0   Fm2
......................................................................ugagcaaaguAucaggugugacu.................   1     1   Fm2
......................................................................ugagcaaaguuucaggugugaUu.................   17    1   Fm2

................................augccugaaauuuucgcuuuga........................................................   5     0   Fm1
................................augccugaaauuuucgcuuugac.......................................................   4     0   Fm1
................................augccugaaauuuucgcuuuUacc......................................................   1     1   Fm1
................................augccugaaauuuucgAuuugacc......................................................   1     1   Fm1
................................augccugaaauuuucgcuuugacc......................................................   11    0   Fm1
................................augccugaaauuuucgcuuugaccG.....................................................   4     1   Fm1
................................augccugaaauuuucgcuuugaccu.....................................................   42    0   Fm1
................................augccugaaauuuucgcGuugaccu.....................................................   1     1   Fm1
................................augccugaaauuuucgcuuugaccuU....................................................   6     1   Fm1
...................................................................Gggugagcaaaguuucaggugugа...................   3     1   Fm1
....................................................................Cgugagcaaaguuucagguguga...................   3     1   Fm1
.....................................................................gugagcaaaguuucaggu.......................   17    0   Fm1
.....................................................................Augagcaaaguuucaggug......................   1     1   Fm1
.....................................................................Nugagcaaaguuucaggug......................   1     1   Fm1
.....................................................................gugagcaaaguuucagguA......................   7     1   Fm1
.....................................................................gugagcaaaguuucagguU......................   4     1   Fm1
.....................................................................gugagcaaaguuucaggCg......................   4     1   Fm1
.....................................................................gugagcaaaguuucaggug......................   156   0   Fm1
.....................................................................gugagcaaaguuucaggAg......................   1     1   Fm1
.....................................................................gugagcaaaguuucaggCgu.....................   3     1   Fm1
.....................................................................gugagcaaaguuucaggugu.....................   217   0   Fm1
.....................................................................gugagcaaaguuucagAugu.....................   1     1   Fm1
.....................................................................gugagcaaaguuucaggugG.....................   19    1   Fm1
.....................................................................guUagcaaaguuucaggugu.....................   1     1   Fm1
.....................................................................gugagcaaaguuucaggGgu.....................   2     1   Fm1
.....................................................................gugagcaaaguuucaggugA.....................   3     1   Fm1
.....................................................................gugagcaaaguuucaggugGg....................   1     1   Fm1
.....................................................................gugagcaaaguuucaggAgug....................   3     1   Fm1
.....................................................................gugagcaaaguuucagguguU....................   10    1   Fm1
.....................................................................gugagcaaaguuucagguguA....................   4     1   Fm1
.....................................................................gugagcUaaguuucaggugug....................   1     1   Fm1
.....................................................................gugagcaaaguuucaggCgug....................   4     1   Fm1
.....................................................................gugagcaaaguuucagAugug....................   1     1   Fm1
.....................................................................gugagcaaaguuuGaggugug....................   1     1   Fm1
.....................................................................gugagcaaaguuucaggugug....................   512   0   Fm1
.....................................................................gugagcaaagAuucaggugug....................   3     1   Fm1
.....................................................................gugagcaaagGuucaggugug....................   1     1   Fm1
.....................................................................gugagcaGaguuucaggugug....................   1     1   Fm1
.....................................................................gugagcaaaguuuAaggugug....................   1     1   Fm1
.....................................................................Uugagcaaaguuucaggugug....................   1     1   Fm1
.....................................................................gugCgcaaaguuucaggugug....................   1     1   Fm1
.....................................................................gCgagcaaaguuucaggugug....................   1     1   Fm1
.....................................................................gugGgcaaaguuucaggugug....................   1     1   Fm1
.....................................................................gugagcaaaguuucGggugug....................   1     1   Fm1
.....................................................................gugagcaaaguuucaggGgug....................   4     1   Fm1
.....................................................................guAagcaaaguuucaggugug....................   1     1   Fm1
.....................................................................Augagcaaaguuucagguguga...................   5     1   Fm1
.....................................................................gugagcaaaguuucaggAguga...................   72    1   Fm1
.....................................................................gugagGaaaguuucagguguga...................   1     1   Fm1
.....................................................................gugagcaaaguAucagguguga...................   1     1   Fm1
.....................................................................gCgagcaaaguuucagguguga...................   4     1   Fm1
.....................................................................Uugagcaaaguuucagguguga...................   12    1   Fm1
.....................................................................gugagcaaaguuGcagguguga...................   1     1   Fm1
.....................................................................gugagcaaaguuucaggGguga...................   65    1   Fm1
.....................................................................gugagcaaaguuucaggugugG...................   5013  1   Fm1
.....................................................................gugagcaaCguuucagguguga...................   2     1   Fm1
.....................................................................gugagcaaaguuucaggugugC...................   62    1   Fm1
.....................................................................gugagcaaaguuucagUuguga...................   21    1   Fm1
.....................................................................gAgagcaaaguuucagguguga...................   7     1   Fm1
.....................................................................gugagcaaagGuucagguguga...................   13    1   Fm1
```

```
                                Star                                Mature
uaggaguguaucuugacuugccguuguugguuaugccugaaauuuucgcuuugaccugacaagggauaggugagcaaaguuucaggugugacugccagguggccccaacac
.....................................................................Nugagcaaaguuucagguguga....................    5      1    Fm1
.....................................................................gugagAaaaguuucagguguga....................    1      1    Fm1
.....................................................................guAagcaaaguuucagguguga....................    3      1    Fm1
.....................................................................gugagcaaaguuuAagguguga....................    4      1    Fm1
.....................................................................gugUgcaaaguuucagguguga....................    2      1    Fm1
.....................................................................gugGgcaaaguuucagguguga....................    3      1    Fm1
.....................................................................guCagcaaaguuucagguguga....................    1      1    Fm1
.....................................................................gugagcaCaguuucagguguga....................    1      1    Fm1
.....................................................................gugaCcaaaguuucagguguga....................    1      1    Fm1
.....................................................................gugagcaaagAuucagguguga....................    23     1    Fm1
.....................................................................gugagcaaaguuucaggugCga....................    2      1    Fm1
.....................................................................gugaAcaaaguuucagguguga....................    3      1    Fm1
.....................................................................gugCgcaaaguuucagguguga....................    4      1    Fm1
.....................................................................gugagcUaaguuucagguguga....................    3      1    Fm1
.....................................................................gugagcaaaguuucagAuguga....................    33     1    Fm1
.....................................................................gugagcaaaguuucagguguga....................    12695  0    Fm1
.....................................................................gugagcaGaguuucagguguga....................    1      1    Fm1
.....................................................................gugagNaaaguuucagguguga....................    1      1    Fm1
.....................................................................gugagcaaaguuucagCuguga....................    36     1    Fm1
.....................................................................gGgagcaaaguuucagguguga....................    2      1    Fm1
.....................................................................gugagcaaaguuCcagguguga....................    1      1    Fm1
.....................................................................gugagcaaagCuucagguguga....................    13     1    Fm1
.....................................................................gugagcaaaguuucagguAuga....................    1      1    Fm1
.....................................................................gugagUaaaguuucagguguga....................    2      1    Fm1
.....................................................................gugagcaaGguuucagguguga....................    1      1    Fm1
.....................................................................gugagcaaaguuuUagguguga....................    10     1    Fm1
.....................................................................guUagcaaaguuucagguguga....................    5      1    Fm1
.....................................................................gugagcNaaguuucagguguga....................    1      1    Fm1
.....................................................................gugagcaUaguuucagguguga....................    1      1    Fm1
.....................................................................gugagcaaaguuucaggCguga....................    83     1    Fm1
.....................................................................gugagcaaaguCucagguguga....................    1      1    Fm1
.....................................................................gugagcaaaguuucUgguguga....................    1      1    Fm1
.....................................................................gugagcaaaguuucGgguguga....................    7      1    Fm1
.....................................................................gugagcaaaguuucaggugAga....................    2      1    Fm1
.....................................................................gugagcaaaguuAcagguguga....................    1      1    Fm1
.....................................................................gugagcaaaguuucagguguUa....................    2      1    Fm1
.....................................................................gugagcaaaguuucagguguCa....................    1      1    Fm1
.....................................................................gugagcaaaCuuucagguguga....................    1      1    Fm1
.....................................................................gugagcaaaguuucaggugugU....................    478    1    Fm1
.....................................................................gugaUcaaaguuucagguguga....................    1      1    Fm1
.....................................................................gugagcGaaguuucagguguga....................    4      1    Fm1
.....................................................................gugagcaaaUuuucagguguga....................    1      1    Fm1
.....................................................................gugagcaaUguuucaggugugac...................    3      1    Fm1
.....................................................................gugagcaaaguuucaggugugaU...................    617    1    Fm1
.....................................................................guAagcaaaguuucaggugugac...................    1      1    Fm1
.....................................................................gugagcaaaguuucaggugugaG...................    29     1    Fm1
.....................................................................gugagcaaaguuucaggugugaA...................    847    1    Fm1
.....................................................................gugagcaaaguuucaggugugCc...................    1      1    Fm1
.....................................................................gugagcaaaguuucaggugugac...................    78     0    Fm1
.....................................................................gugagcaaaguuucaggugugacu..................    9      0    Fm1
.....................................................................gugagcaaaguuucaggugugaGu..................    11     1    Fm1
.....................................................................gugagcaaaguuucaggugugacC..................    12     1    Fm1
.....................................................................gugagcaaaguuucaggugugaAu..................    46     1    Fm1
.....................................................................gugagcaaaguuucaggugugaUu..................    85     1    Fm1
.....................................................................gugagcaaaguuucaggugugacCg.................    1      1    Fm1
......................................................................ugagcaaaguGucagguguga....................    1      1    Fm1
......................................................................ugagcaaaguuucagguguga....................    141    0    Fm1
......................................................................ugagcaaaguuucaggugugG....................    51     1    Fm1
......................................................................ugagcaGaguuucagguguga....................    1      1    Fm1
......................................................................ugagcaaaguuucUgguguga....................    1      1    Fm1
......................................................................ugagcaaaguuucaggugugU....................    13     1    Fm1
......................................................................ugagcaaaguuucaggAguga....................    2      1    Fm1
......................................................................ugagcaaaguuucaggugugaU...................    17     1    Fm1
......................................................................ugagcaaUguuucaggugugac...................    7      1    Fm1
......................................................................ugagcaaaguuucaggugugac...................    24     0    Fm1
......................................................................ugagcaaaguuucaggugugaA...................    14     1    Fm1
......................................................................ugagcaaaguuucaggugugacu..................    3      0    Fm1
......................................................................ugagcaaaguuucaggugugaUu..................    4      1    Fm1
......................................................................ugagcaaaguuucaggugugacuU.................    5      1    Fm1
.......................................................................gagcaaaguuucaggugug.....................    3      0    Fm1
```

Star **Mature**

uaggaguguaucuugacuugccguuguugguuaugccugaaauuuucgcuuugaccugacaagggauaggugagcaaaguuucaggugugacugccagguggccccaacac
